# Human fetal neuroblast and neuroblastoma transcriptome analysis confirms neuroblast origin and highlights neuroblastoma candidate genes

**DOI:** 10.1186/gb-2006-7-9-r84

**Published:** 2006-09-21

**Authors:** Katleen De Preter, Jo Vandesompele, Pierre Heimann, Nurten Yigit, Siv Beckman, Alexander Schramm, Angelika Eggert, Raymond L Stallings, Yves Benoit, Marleen Renard, Anne De Paepe, Geneviève Laureys, Sven Påhlman, Frank Speleman

**Affiliations:** 1 Center for Medical Genetics, Ghent University Hospital, De Pintelaan, B-9000 Ghent, Belgium; 2 Department of Medical Genetics, University Hospital Erasme, Lenniksebaan, B-1070 Brussels, Belgium; 3 Division of Molecular Medicine, Department of Laboratory Medicine, Lund University, University Hospital MAS, SE-20502 Malmö, Sweden; 4 Department of Pediatric Oncology and Hematology, University Hospital of Essen, Hufelandstr, Essen 45122, Germany; 5 Children's Cancer Research Institute, University of Texas Health Science Center, Floyd Curl Drive, Mail Code 7784, San Antonio, Texas 78229-3900, USA; 6 Department of Pediatrics, Ghent University Hospital, De Pintelaan, B-9000 Ghent, Belgium; 7 Department of Pediatrics, UZ Gasthuisberg, Herestraat, B-3000 Leuven, Belgium

## Abstract

**Background:**

Neuroblastoma tumor cells are assumed to originate from primitive neuroblasts giving rise to the sympathetic nervous system. Because these precursor cells are not detectable in postnatal life, their transcription profile has remained inaccessible for comparative data mining strategies in neuroblastoma. This study provides the first genome-wide mRNA expression profile of these human fetal sympathetic neuroblasts. To this purpose, small islets of normal neuroblasts were isolated by laser microdissection from human fetal adrenal glands.

**Results:**

Expression of catecholamine metabolism genes, and neuronal and neuroendocrine markers in the neuroblasts indicated that the proper cells were microdissected. The similarities in expression profile between normal neuroblasts and malignant neuroblastomas provided strong evidence for the neuroblast origin hypothesis of neuroblastoma. Next, supervised feature selection was used to identify the genes that are differentially expressed in normal neuroblasts versus neuroblastoma tumors. This approach efficiently sifted out genes previously reported in neuroblastoma expression profiling studies; most importantly, it also highlighted a series of genes and pathways previously not mentioned in neuroblastoma biology but that were assumed to be involved in neuroblastoma pathogenesis.

**Conclusion:**

This unique dataset adds power to ongoing and future gene expression studies in neuroblastoma and will facilitate the identification of molecular targets for novel therapies. In addition, this neuroblast transcriptome resource could prove useful for the further study of human sympathoadrenal biogenesis.

## Abstract 2

Transcriptome profiling of neuroblasts and neuroblastoma tumor cells provides strong support for a neuroblast origin of neuroblastoma and highlights new candidate neuroblastoma genes

## Background

Neuroblastoma is the most common and deadly extracranial solid childhood tumor, exhibiting remarkable variation in clinical presentation ranging from localized to highly metastatic disease. Despite multimodal therapies, survival rates for aggressive neuroblastomas are still disappointingly low. One possible approach to development of more efficient and less toxic therapies is to gain insight into the signaling pathways that are deregulated in neuroblastoma and to use this information in the design of molecular therapies. However, at present only two genes, namely *MYCN *and *PHOX2B*, have been directly linked to neuroblastoma development, although their exact role in oncogenesis is still unclear [1,2].

It is hoped that genome-wide gene expression studies will provide insights into the genes and molecular pathways that govern neuroblastoma pathogenesis. Thus far, no clear or consistent candidate genes or pathways have emerged from these analyses [3-5] (see Additional data file 3 for more references). Both for currently available expression data and forthcoming datasets, we anticipate that transcriptome information on the cells of origin of neuroblastoma (sympathetic nervous system progenitors) will be of crucial importance and could provide significant power on data mining strategies.

The sympathetic nervous system is composed of sympathetic chain and truncus ganglia, paraganglia, and the adrenal gland. Ganglion cells (neuroblasts during development) are the major cell type of chain and truncus ganglia, and extra-adrenal chromaffin cells form the paraganglia, whereas the adrenal gland is composed of adrenal chromaffin cells and, at least during development, sympathetic neuroblasts. The fate of the neuroblasts in the developing human adrenal gland is not clear; some or all may involute or mature as solitary intra-adrenal neurons [6]. Evidence for the cellular origin of neuroblastoma is based on their occurrence in the adrenal gland or along the spinal cord in association with sympathetic ganglia, and on their neuroblastic phenotype that indicates that the tumor cells are derived from immature sympathetic nervous system cells of the ganglionic lineage [7]. Indeed, cells of adrenal neuroblastomas have neuroblastic morphology and do not express the adrenal chromaffin marker *PNMT*, but they share phenotypic characteristics with the immature sympathetic neuroblasts present as nests of cells in the developing adrenal gland. However, a small subset of neuroblastomas also contains cells with extra-adrenal chromaffin characteristics.

In the present study we isolated and performed expression profiling of the human adrenal neuroblasts as they form monocellular structures during early fetal stages, which can be easily microdissected. In parallel, favorable and unfavorable neuroblastoma tumors were profiled on the same platform. Finally, our dataset was integrated in a meta-analytical data mining approach.

## Results

### Characterization, isolation, and gene expression profiling of fetal adrenal neuroblasts

**Figure 1:**
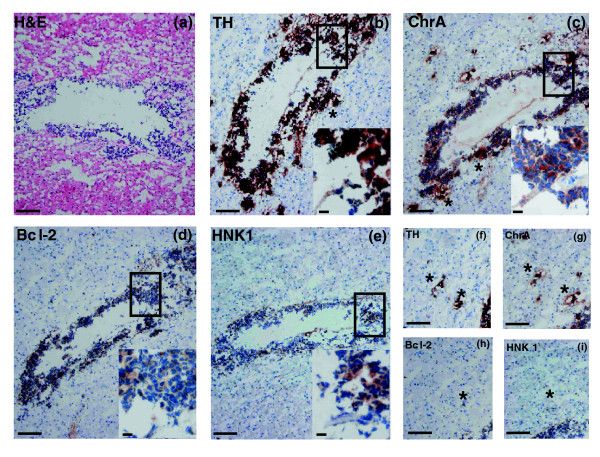
Identification of sympathetic neuroblasts and chromaffin cells in human fetal adrenal glands by immunohistochemical analysis. Sections of a human fetal (19 weeks) adrenal gland, adjacent to those used for laser capture retrieval of cells for mRNA extraction and gene expression profiling, were stained with **(a) **hematoxylin and eosin or antibodies directed against **(b,f) **TH, **(c,g) **CHGA, **(d,h) **BCL2, and **(e,i) **HNK1. Whereas the immunoreactivities of BCL2 and HNK1 are specific for neuroblasts, TH and CHGA expression is pronounced in chromaffin cells and weak in neuroblasts [8]. Stars indicate chromaffin cells (TH^+^, CHGA^+^, BCL2^-^, and HNK1^-^), either solitary or intermingled with neuroblasts. Panels a-e show a cluster of adrenal neuroblasts and panels f-i show cortical area within scattered chromaffin cells adjacent to the neuroblast cluster. Inserts in panels b-e (bars: 10 μm) correspond to the boxed areas in these panels (bars in panels a-i: 100 μm). BCL2, B-cell CLL/lymphoma 2; CHGA, chromogranin A; H&E, hematoxylin and eosin; HNK1, carbohydrate epitope; TH, tyrosine hydroxylase.

Prescreening of hematoxylin-eosin cryosections from 11 fetal adrenal glands demonstrated that large neuroblast clusters of more than 100 cells were predominantly found in adrenal glands at 19 and 20 weeks' gestational age (Figure 1a). To verify that these cell clusters indeed represent neuroblasts and to estimate the degree of intermingled chromaffin cells, cryosections were stained for the neuronal and chromaffin marker TH (tyrosine hydroxylase), the chromaffin marker CHGA (chromogranin A; which also has low expression in neuroblasts), and the neuronal markers BCL2 (B-cell CLL/lymphoma 2) and HNK1 (carbohydrate epitope) [8]. As shown in Figure 1, the clusters of neuroblastic cells stained positive for all markers and, in particular, these cells were positive for BCL2 and HNK1. The majority of chromaffin cells, identified by their strong CHGA and TH expression, were found to be scattered throughout the adrenal cortex (these cells coalesce and form large islands of chromaffin cells later during development), whereas a few cells were located in or adjacent to the neuroblast clusters.

**Figure 2:**
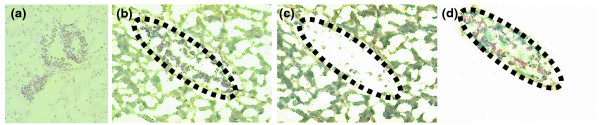
Laser capture microdissection of neuroblast clusters. **(a) **Large cluster of neuroblasts in fetal adrenal glands at 19 weeks' gestational age (mounted hematoxylin and eosin stained cryosections), **(b,c) **unmounted hematoxylin and eosin stained fetal adrenal cryosections with a neuroblast cluster before and after microdissection (sample 2), and **(d) **the microdissected neuroblast cluster.

Neuroblast clusters and adjacent cortical cells (used as controls) were isolated using laser capture microdissection from stained cryosections from three different fetal adrenal glands (glands 1, 2 and 3, which were of gestational ages 20, 19 and 19 weeks, respectively) (Figure 2) and immediately lysed in RNA extraction buffer. In order to obtain a sufficient amount of good quality neuroblast RNA for oligonucleotide chip analyses, we applied a previously validated protocol for tissue sectioning, staining, and microdissection [9] (Additional data file 1(a)). By pooling different isolates of the same adrenal gland, between 2.5 and 15 ng total RNA could be obtained for each of the three neuroblast samples (Additional data file 1(b)). After two-round amplification and labeling of three neuroblast, three cortex, and 18 neuroblastoma RNA samples, hybridization was performed on HG-U133A Affymetrix oligonucleotide chips. Real-time polymerase chain reaction analysis of selected genes showed that there was no RNA amplification bias in the chip data (Additional data file 1(c)).

### Validation of the expression profile of fetal adrenal neuroblasts and cortex cells

The expression profiles of the neuroblast and cortex samples were compared using the rank product nonparametric method, which is particularly suited for extracting significantly differentially expressed genes in a limited number of samples [10]. Two lists of 156 and 86 unique genes were established with significantly higher expression in neuroblast and adrenal cortex cells, respectively (multiple testing corrected *P *< 0.01; Additional data file 2). Gene Ontology (GO) analysis identified those classes of genes that are significantly over-represented in the cell specific gene lists (*P *< 0.01; Table 1). As expected, the neuroblast gene list is enriched for genes that are involved in catecholamine metabolism, neurogenesis and other neural processes, whereas cortex cells specifically express genes involved in steroid and cholesterol metabolism.

To further test the validity of the neuroblast gene expression profile, we evaluated the expression of known neuronal and chromaffin markers that were previously studied in human fetal sections [8]. High expression (among the 10% most abundant genes) of neuronal markers (*BCL2*, *GAP43*, and *NPY*) together with chromaffin (and to a lesser extent neuronal) markers (*CHGA*, *CHGB*, *DBH*, *DDC *and *TH*) and an adrenal chromaffin marker (*PNMT*) in the microdissected cell clusters is in keeping with our observation that the neuroblast isolates are pure, with only rare intermingled chromaffin cells (Figure 1).

Gene set enrichment analysis [11] based on expression of the 156 neuroblast-specific genes in 79 human tissues [12] was performed in order to explore whether the microdissected neuroblasts indeed have neural characteristics. The neuroblasts exhibit a significant overlap in expression with various nervous system tissues (*P *< 0.05; fetal brain, prefrontal cortex, brain amygdale, whole brain, occipital lobe, and hypothalamus), further demonstrating that the proper cells were microdissected.

### Similarity between the expression profiles of neuroblast and neuroblastoma further supports the 'cell of origin' concept

Although multiple lines of evidence indicate that neuroblastoma originates from immature sympathetic neuroblasts, the mRNA expression repertoire of these neuroblasts and neuroblastomas have not yet been compared. Before our analysis, we assumed that, in addition to differences resulting from oncogenic transformation, both cell populations would exhibit many cell type specific similarities.

**Figure 3:**
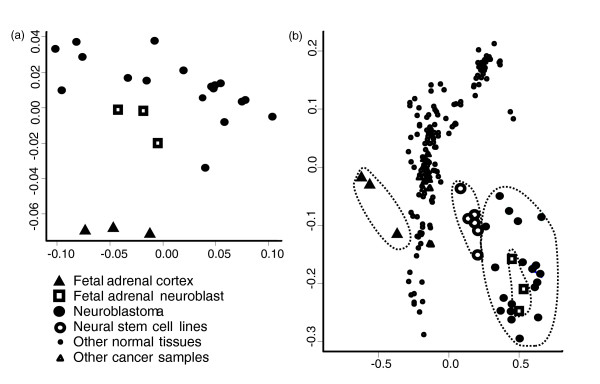
Multidimensional scaling of neuroblast, cortex, and neuroblastoma samples. **(a) **Multidimensional scaling of neuroblast, cortex, and neuroblastoma samples using all genes (Spearman correlation) and **(b) **multidimensional scaling of neuroblast, cortex, neuroblastoma, 79 normal tissue samples and other cancer samples (in duplo), and three neural stem cell cultures using the genes that are differentially expressed between fetal adrenal neuroblast and fetal adrenal cortex shows that the neuroblasts cluster very close to the neuroblastomas.

Three data mining strategies were employed to investigate this hypothesis. First, an unbiased multidimensional scaling of all genes on the chip showed that the neuroblasts cluster close to the neuroblastoma tumors and that both groups cluster far away from the fetal adrenal cortex cells (Figure 3a). Second, we extended our dataset with publicly available expression profiles (measured on the same platform) from 79 normal tissues [12]and three neural stem cell cultures [13]. Based on the genes that are differentially expressed between the neuroblasts and cortex samples (156 and 86 genes, respectively), multidimensional scaling showed again that the neuroblastoma tumors cluster close to the neuroblasts and further away from the other normal tissues. Interestingly, the neural stem cells also cluster close to the neuroblastomas and neuroblasts (Figure 3b). These findings further support the notion that adrenal neuroblasts are indeed of neuronal origin with possible neuronal stem cell features, and the observed considerable similarities to neuroblastomas in terms of expression give further strength to the 'cell of origin' hypothesis for neuroblastoma development.

**Figure 4:**
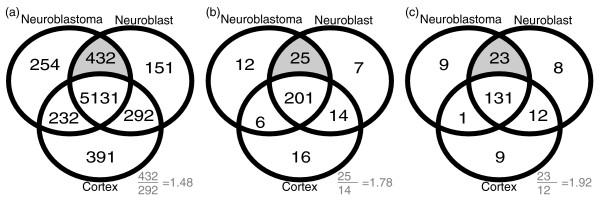
Venn diagram analysis of the genes with detectable expression in neuroblastoma, neuroblast, and cortex samples. **(a) **All genes, **(b) **transcription factors (GO:0003700), and **(c) **neurogenesis genes (GO:0007399). The number of genes that are in common between neuroblast and neuroblastoma is higher than the number of genes that are in common between the neuroblasts and cortex samples (especially for the gene classes transcription and neurogenesis), indicating that neuroblastomas resemble neuroblasts. GO, Gene Ontology.

Third, we looked for similarities in mRNA expression between neuroblast and neuroblastoma by cataloging their expression repertoire. We defined a reasonable cut-off to determine whether a gene is expressed or not in a given sample (the mean percentage of present calls for the various chips; Additional data file 1(d)). As such, the 36% most highly expressed probe IDs in the cortex, neuroblast and neuroblastoma cells, were selected and compared in a Venn diagram. This analysis clearly shows that neuroblasts have more expressed genes in common with neuroblastoma than with the cortex cells (432 versus 292; Figure 4a). GO analysis on the common 432 genes revealed an expected over-representation of neurogenesis genes (*P *< 0.01; data not shown). Next, we zoomed in on neurogenesis and transcription factor ontology classes by performing a similar Venn diagram for these gene sets, assuming their putative importance in neuroblastoma development. Interestingly, the similarities between neuroblast and neuroblastoma are even more pronounced for these two GO classes (Figure 4b, c).

### Identifying genes and pathways putatively implicated in neuroblastoma pathogenesis through differential expression analysis of normal neuroblasts and neuroblastomas

In the final and most challenging part of our data mining approach, we aimed to identify genes that are under-expressed or over-expressed in neuroblastomas compared with neuroblasts, because these genes and the pathways that they govern might be involved in neuroblastoma development or represent markers for the stage of developmental arrest of neuroblastomas. Rank product analysis (multiple testing corrected *P *< 0.01) yielded a list of 71 genes that were more highly expressed in neuroblasts and 565 genes that were more highly expressed in neuroblastomas (Additional data file 2).

A first crucial step in our data mining strategy to identify genes that are putatively involved in neuroblastoma was a meta-analysis of our generated gene lists in published neuroblastoma microarray data. We used the Neuroblastoma Gene Server (NBGS) which was developed in-house (see Additional data file 3 for detailed information) to compare the neuroblast-specific and neuroblastoma-specific gene lists with genes that have been reported as differentially expressed in 25 previous gene expression profiling studies conducted in neuroblastoma (Additional data file 3). We found that as many as 17 of the 71 genes (24%) that are over-expressed in neuroblasts relative to neuroblastomas were reported in the NBGS, mainly annotated as genes that are more highly expressed in maturing, differentiating, or localized neuroblastomas. Likewise, 102 out of the 565 genes (18%) that were over-expressed in neuroblastoma were previously identified in other gene expression studies on neuroblastoma. The high overlap of our gene lists with published gene lists demonstrates the validity of our lists, which were subsequently further explored in chromosomal mapping, GO, and pathway analysis.

### Positional expression mapping of candidate oncogenes and tumor suppressor genes

**Figure 5:**
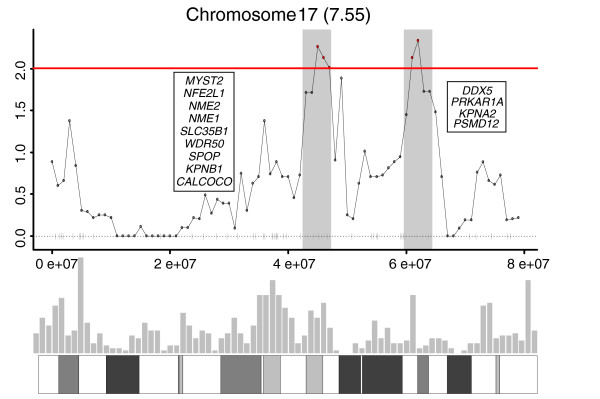
Positional gene enrichment analysis of genes on chromosome 17. Positional gene enrichment analysis for the genes that are more highly expressed in neuroblastoma compared to normal neuroblasts identified two regions on 17q with significant over-representation (-^10^log *P *values; indicated in grey; the genes in these regions are printed in the boxes). The horizontal red line indicates the multiple testing corrected *P *value of 0.01, above which the positional gene enrichment value denotes significant over-representation. Vertical lines show the position of the genes on chromosome 17 from the gene list under investigation. The boxplot shows the gene density along the chromosome.

Chromosome 17q gain is the most frequent genetic aberration in neuroblastoma and is assumed to play a crucial role in its pathogenesis through a dosage effect of one or more genes. This critical region still comprises 25 megabases (Mb) [14], precluding straightforward candidate gene identification. Here we apply an alternative, intuitive strategy to pinpoint putative critical dosage sensitive loci. Using positional gene enrichment analysis (De Preter and coworkers, unpublished data) [15], we sought chromosomal loci that are significantly over-represented in the list of genes that are over-expressed in neuroblastoma relative to their normal cells of origin (Figure 5). We found two peaks on chromosome 17q, with high significance for a locus on 17q21.32-q22 that coincides with the consistently gained segment just distal from the most distal breakpoint in a series of high-resolution copy number profiles (Vandesompele and coworkers, unpublished data).

Apart from over-expressed genes, we also sought positional tumor suppressor genes by mapping under-expressed genes (relative to normal neuroblasts). The following positional candidates could be identified, located within or very close to the known shortest regions of overlap in neuroblastoma: *CASP9 *on 1p36; *CACNA2D3*, *TDGF1 *and *NKTR *on 3p21-p22 (SRO (shortest region of overlap) from [16]); *IGSF4*, *APOA1*, *MLL *and *RDX *on 11q23 [17-19]; and *MEG3 *and *DLK1 *on 14q32 [20].

### GO analysis

To examine gene expression differences between neuroblasts and neuroblastomas from a different perspective, we mapped the neuroblast-specific and neuroblastoma-specific gene lists to the biologic process GO classification (Table 2). This revealed that the neuroblasts express significantly (*P *< 0.01) more genes that are involved in steroid and catecholamine metabolism compared with neuroblastomas. Neuroblastomas are characterized by an over-representation of genes that are involved in immune response, cell growth, and cell cycle. The immune response gene signature may be due to infiltrating immune cells, whereas the over-representation of cell growth and cell cycle genes in neuroblastomas is in perfect concordance with the hyperproliferative character of tumors.

We then specifically looked at genes belonging to GO terms neurogenesis, transcription factor activity, and apoptosis; these three processes can be assumed to play an important role in neuroblastoma pathogenesis (Table 3). This analysis identified the following interesting genes from the neuroblast-specific and neuroblastoma-specific gene lists: transcription factors involved in neurogenesis *TFAP2B *(6p12.3; more highly expressed in neuroblasts); *ASCL1 *(12q23.2), *SIX3 *(2p21) and *STAT3 *(17q21.2; more highly expressed in neuroblastoma); and *APOE *(19q13.31) and *INHBA *(7p14.1; more highly expressed in neuroblastoma), which are involved in both apoptosis and neurogenesis.

### Differential expression analysis of favorable and unfavorable neuroblastomas

Thus far, most published microarray studies on neuroblastomas mainly compared favorable with unfavorable neuroblastomas in order to identify prognostic markers or pathways that are involved in these clearly different neuroblastoma tumor types. In order to add value to such an analysis, we contrasted similar differentially expressed gene lists with the normal neuroblast expression profile (Additional data file 2). In a first step, we compared the differentially expressed genes between these two tumor types with published prognostic gene lists. We found that 25 of the 194 genes on our list were previously reported, including the well established markers *MYCN*, *NTRK1*, and *CD44 *(see NBGS analysis in Additional data file 3). This overlap demonstrates the validity of the selected neuroblastoma panel and their expression profile. Subsequently, we sought the corresponding gene expression levels of the differentially expressed genes in the normal counterpart cells, aiming to select neuroblastoma candidate genes. Of the 95 genes that are more highly expressed in favorable tumors (versus unfavorable ones), 37 also have significant differential expression (either higher or lower) compared with neuroblasts, whereas 41 out of the 101 genes that are more highly expressed in unfavorable tumors exhibit differential expression compared with the neuroblasts (Table 4).

From this analysis, a few putative positional tumor suppressor candidates emerge: *CDC42 *on 1p36, *CACNA2D3 *on 3p21, and *DLK1 *on 14q. The latter two genes are of particular interest because they are highly expressed in neuroblasts and favorable neuroblastomas, and their expression is significantly lower in unfavorable neuroblastomas. Among the genes that are more highly expressed in unfavorable neuroblastomas than in favorable ones and neuroblasts, the proven oncogenic transcription factor *MYCN *emerges (and putative downstream genes *KIFAP3*, *OPHN1, RGS7*, *ODC1*, *TOP2A*, *TWIST1 *and *TYMS*, according to NBGS*)*, as do several other genes that have been identified or studied within the context of neuroblastomas such as *ALK *and *PRAME*, and positional candidates on 17q including *BIRC5*, *RNU2 *and *TOP2A*.

### Expression of neurogenesis markers in neuroblasts and developmental origin of neuroblastoma

Although this was not the primary aim of the present work, the neuroblast expression profile provides a unique resource for the investigation of gene expression in human sympatho-adrenal progenitors. In a first attempt, we made an inventory of the genes that belong to the neurogenesis GO class, or that have been described to play a role in neural crest formation and migration, or that have proneural activity (Additional data file 4). This analysis showed that human fetal neuroblasts of 19 weeks' gestational age expressed 174 of the 359 genes in the neurogenesis GO class, and 26 of 89 proneural genes and genes involved in neural crest formation/migration.

**Figure 6:**
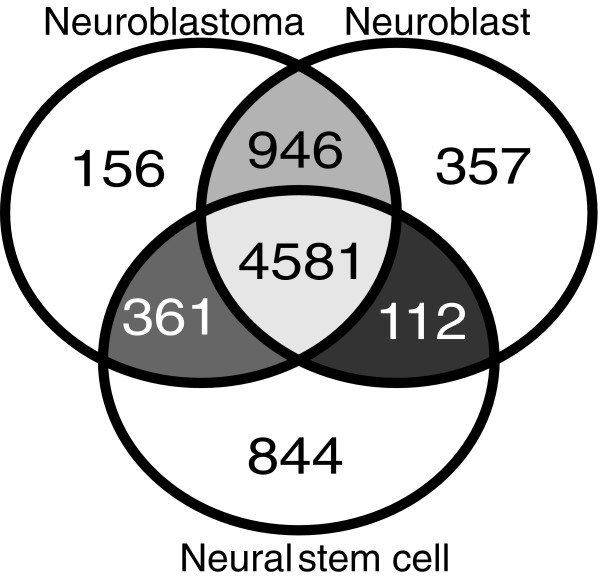
Venn diagram analysis of genes with detectable expression in neuroblast, neuroblastoma, and neural stem cell lines. This analysis shows that neuroblasts have many genes in common with neuroblastoma, but it also demonstrates that neural stem cell lines have more genes in common with the neuroblastomas than with the normal neuroblasts.

To obtain possible clues on the developmental origin of neuroblastoma we compared the expression profiles of the neuroblastoma tumors with those of normal neuroblasts and neural stem cell cultures. Intersectional Venn diagram analysis of expressed genes shows that neuroblastomas have many genes in common with neuroblasts, as already shown above (Figure 6). Interestingly, when compared with neuroblasts, the neuroblastomas have more genes in common with the self-renewing neural stem cells (535 versus 145), among others the neurogenesis genes *ASCL1*, *GSS*, *STAT3*, *UTP11L*, *ENAH*, *APBB2*, *CDK5RAP2*, and *LARGE*.

## Discussion

Comparison of the mRNA expression repertoire of cancer with that of their normal counterpart cells is a commonly applied strategy to elucidate the development and pathophysiology of the cancer type under study. For pediatric neuroblastoma, fetal adrenal sympathetic neuroblasts are assumed to be the cells of origin, but these cells are virtually absent after birth and thus not readily accessible for analysis [8]. In this study we were able, for the first time, to determine the expression profile of microdissected islets of fetal sympathetic neuroblasts, providing an important landmark for comparative expression analysis. In parallel, adjacent cortex cells and carefully selected representative neuroblastoma tumors were profiled for data mining purposes. Our main goals were to provide support for the cell of origin hypothesis of neuroblastoma, and to obtain preliminary insights into the disrupted cellular circuitry that is involved in neuroblastoma pathogenesis.

Quality assessment and biologic validation of the established neuroblast expression profile demonstrated that the proper cells were isolated and that their expression profiles are trustworthy. Next, we assessed the cell of origin hypothesis for neuroblastomas. To this end, the transcriptional profile of the neuroblasts was thoroughly compared with those of neuroblastoma tumors and normal tissues. These analyses confirmed that neuroblast and neuroblastoma cells indeed present with highly similar expression profiles. These exploratory findings provide, for the first time, molecular support for the cell of origin hypothesis. Also, they reinforce our assumption that the neuroblast gene expression profile constitutes a valid tool for further data mining of neuroblastoma gene expression patterns.

Following initial data validation and assessment of the cell of origin hypothesis, we performed a series of data mining analyses aimed at identifying genes and pathways that may be involved in neuroblastoma oncogenesis and tumor biology. In a first step, the neuroblastoma tumor expression profile was compared with that of the neuroblasts, yielding 71 genes with higher expression in neuroblasts and 565 genes with increased expression in neuroblastoma. We subjected these gene lists to a novel meta-analytical approach that allowed comparison with 25 published neuroblastoma gene lists and facilitated the detection of genes identified in at least one other microarray study. Furthermore, we performed GO analysis and we used a new approach to positional mapping of the differentially expressed genes. In a second step, following analysis of combined tumors, we sought genes differentially expressed in carefully selected representative cases of favorable and unfavorable neuroblastomas and further analyzed the expression of these genes in neuroblasts. This approach yielded 37 and 41 genes, respectively.

When combining the data from the above analyses, it was apparent that many of the genes previously reported in the context of neuroblastoma had been identified, thus underscoring the validity of our data mining approach. These included *MYCN*, *MYCN *co-amplified genes such as *DDX1*, known *MYCN *target genes such as *ODC1 *and *MCM7*, and prognostic markers (*MYCN*, *NTRK1*, and *CD44*), as well as various other genes such as *ASCL1*, *ALK*, *BCL2*, *BIRC5*, *DLK1*, *NME1*, *NME2 *and *NTRK3 *that have previously been mentioned or studied within the context of neuroblastoma. For some genes only circumstantial evidence for a role in neuroblastoma is present (*WSB1*, *CDC42*, *PLAGL1*, *PRAME *and *TGFBR3*); that we identified these genes in the present study warrants further investigations into their possible role in neuroblastoma development. Finally, several genes, for which no evidence of involvement in neuroblastoma development has yet been obtained, emerged for the first time from our analyses. These include *STAT3*, *IGSF4 *and *CACNA2D3*, and they should also be studied in further detail to determine their possible role in neuroblastoma pathogenesis.

Although the present study is just a first step in a new strategy of data mining of neuroblastoma gene expression profiles, we nevertheless obtained new information that is particularly interesting for the 17q region. Gain of distal 17q is not only the most frequent chromosomal alteration in high stage neuroblastoma but it is also the strongest independent adverse prognostic genetic factor [21,22]. However, no functional evidence has been provided for a specific role of 17q genes in neuroblastoma development. A major obstacle is the difficulty in refining the critical region for 17q gain that, as a consequence, has remained very large, hampering selection of functional candidates. Based on recent high-resolution array-CGH (comparative genome hybridization) profiling of 17q breakpoints leading to gain for distal 17q, we have proposed the hypothesis that the critical region for 17q gain is located within a 5 Mb segment on 17q21.32-q22, immediately distal to the most distal breakpoint (Vandesompele and coworkers, unpublished data).

To substantiate this hypothesis, we performed positional gene enrichment analysis on chromosome 17 for the genes that are more highly expressed in neuroblastoma compared to neuroblast. Interestingly, this yielded a highly significant enrichment for two loci on the long arm of chromosome 17, including the above mentioned region, further demonstrating the high likelihood of the presence of a neuroblastoma dosage sensitive gene. A total of 11 differentially expressed genes are contained within this 17q21.3 segment, including *NME1 *and *NME2*. The role of the latter two genes in cancer is controversial, but once again these genes emerge from a neuroblastoma study. Among the genes that are more highly expressed in neuroblastoma, another interesting candidate was found to be located just outside the enriched 17q21.3 segment but within the same chromosome band, namely *STAT3*. This gene encodes an oncogenic transcription factor that plays a central role in the janus kinase (JAK)-signal transducer and activator of transcription (STAT) signaling pathway, promoting growth and survival of tumor cells, inducing tumor angiogenesis, and suppressing antitumor immune responses. Of particular interest is that *STAT3 *is also implicated in neurogenesis. Given their documented role in cancer, STAT proteins have been shown to be promising molecular targets for novel cancer therapies, including small molecule inhibitors of STAT signaling. The finding of increased *STAT3 *expression might also be of relevance in the light of the observed *ALK *over-expression in this and previous studies [23,24], because ALKis known to activate STAT3 by phosphorylation [25]. Suppression of activated ALK in neuroblastoma cells by RNA interference was shown to lead to rapid apoptosis [26].

Positional mapping of the genes that are expressed to a lesser degree in neuroblastomas than in neuroblasts yielded some remarkable positional tumor suppressor candidate genes. Among others, these include *CASP9 *and *CDC42 *(1p36), which have already been studied in neuroblastoma [27,28]; *CACNA2D3 *(3p21-p22), which was recently proposed as a tumor suppressor gene in lung cancer [29]; *IGSF4 *(11q23), which is a known tumor suppressor gene in several cancers; and *DLK1 *(14q). All of these genes have been mapped within or near to previously defined shortest regions of overlap for deletions in neuroblastoma and should therefore be considered for further functional studies.

Yet another interesting candidate neuroblastoma suppressor gene is *WSB1*. This gene was found in four published neuroblastoma microarray studies to be more highly expressed in favorable neuroblastomas. Moreover, *WSB1 *was very recently shown to be associated with prognosis [30]. Recent evidence indicated that WSB1 (WD repeat and SOCS box-containing 1) is part of an E3 ubiquitin ligase and that it exhibits similarity with an interchangeable F-box protein β-TrCP1 that is implicated in nuclear factor-κB, Wnt/Wingless, and hedgehog signaling pathways [31,32]. Together with other unpublished data on the possible implications of the Wnt pathway in neuroblastomas, we speculate that reduced ubiquitination of β-catenin caused by low levels of *WSB1 *expression in unfavorable neuroblastomas could lead to upregulation of several genes that are involved in cell proliferation [33].

Finally, *PLAGL1 *was identified as a candidate neuroblastoma tumor suppressor gene in the present study. This gene regulates apoptosis and cell cycle arrest and plays a role in the control of cell fate during neurogenesis [34]. *PLAGL1 *is localized on chromosome 6q24-q25, a region that is frequently deleted or epigenetically modified in many solid tumors [35], including neuroblastoma (unpublished data).

The dataset presented will also be of future value for the study of sympathetic nervous system development and the developmental stage from which neuroblastoma originates. Ideally, more neuroblast samples from different gestation times should be collected in order to gain broader insight. The present neuroblast collections offer a glimpse into this developmental process, as illustrated by the expression of *ASCL1 *and *DLK1*. *ASCL1 *is a known early neurogenesis marker [36], which was confirmed by the observed expression in the immature self-renewing neural stem cells and the absence in the more mature neuroblasts. The significantly higher expression in part of the unfavorable neuroblastomas compared with the neuroblasts might denote an earlier stage of differentiation arrest or reflect a process of de-differentiation of the unfavorable neuroblastoma cells. *DLK1*, on the other hand, is expressed to a lesser degree in the unfavorable neuroblastoma than in the favorable tumors and the neuroblast (in concordance with observations reported by Hsiao and coworkers [37]). Later in neural development, DLK1 (delta-like 1 homolog) downregulates ASCL1 (achaete-scute complex-like 1) through NOTCH (notch homolog), further inducing neuronal differentiation [38]. Hence, these expression differences indicate a different time point of developmental arrest for favorable and unfavorable neuroblastoma, as was previously suggested.

## Conclusion

The inclusion of normal neuroblasts in gene expression analysis of malignant neuroblastomas was shown to add significant power to the identification of candidate neuroblastoma genes. Inclusion of larger sets of neuroblastoma tumors with well characterized genomic alterations and positional mapping of the genes in critically involved genomic regions in neuroblastomas will be crucial for tracing back the molecular basis of neuroblastoma.

## Materials and methods

### Fetal and tumor material

Ethical approval was obtained for the collection of fetal adrenal glands from fetuses aborted for clinical reasons (Ethics committee Erasme Hospital, Brussels, Belgium; approval no.: OM021). The induced abortion was performed by prostaglandin instillation to the patient. The adrenals were removed during necropsy and snap-frozen in liquid nitrogen within 3 hours after delivery. Neuroblastoma tumors were collected in the Center for Medical Genetics (Ghent, Belgium; *n *= 12), in the National Center for Medical Genetics (Dublin, Ireland; *n *= 1), and in the University Children's Hospital of Essen (Essen, Germany; *n *= 5). For this study, we preferentially selected tumors that were localized in the adrenal gland (11/18). Based on INSS stage (international neuroblastoma staging system), *MYCN *status, ploidy and age at diagnosis, and for some cases pathologic rapports, samples were divided into favorable or unfavorable neuroblastoma (Table 5).

### Hematoxylin and eosin staining, immunohistochemistry, and laser capture microdissection

Fetal adrenal glands were embedded in Tissue-Tek OCT compound (Sakura, Torrance, CA, USA). Immunohistochemical staining was performed as described previously [8]. For microdissection, cryosections were first stained with hematoxylin and eosin, and mounted in order to scan for neuroblast clusters. When neuroblast clusters were found, stained but unmounted cryosections were prepared for laser capture microdissection. Embedding, sectioning, staining, and laser capture microdissection of neuroblast clusters and surrounding cortex cells was performed as described previously [9].

### RNA isolation and quality assessment

Microdissected cells were collected in RNA extraction buffer, followed by RNA extraction and DNase treatment on column (Qiagen, Venlo, Netherlands). RNA of the tumor samples was extracted using the RNeasy Mini kit (Qiagen), in accordance with the manufacturer's instructions. Four of the neuroblastoma tumor pieces were first mixed with Lysing Matrix D microbeads (Qbiogene, Illkirch, France) and 700 μl RTL buffer (Qiagen), and homogenized using FastPrep FP220 (Qbiogene). A fraction of the RNA was used for cDNA synthesis after DNase treatment (described by Vandesompele and coworkers [39]). RNA quality was measured with the RNA Nano or Pico LabChip kit (Agilent, Diegem, Belgium) using 1 μl of the RNA isolates.

### Oligonucleotide chip analysis and data mining

For each of the three fetal adrenal glands, the different neuroblast RNA isolates were pooled, amplified using a two-round labeling protocol, and hybridized to HG-U133A oligonucleotide chips (Affymetrix, Santa Clara, CA, USA), containing 18,400 transcripts including 14,500 well characterized human genes (protocol described previously [40]). The same amplification protocol was applied to RNA of three cortex samples and approximately 100 ng RNA of 18 neuroblastoma tumors. The homogeneity of the subgroups (neuroblast, cortex, favorable and unfavorable neuroblastoma) allowed us to use a limited number of samples for expression profiling. Several technical parameters demonstrate that the hybridization was of good quality (Additional data file 1(d)).

We obtained the raw data from the Genomics Institute of the Novartis Foundation compendium of normal tissues consisting of 79 normal tissues assayed in duplicate using the Affymetrix HG-U133A array [12]. Raw HG-U133A Affymetrix array data from three neural stem cells were kindly provided by Wright and coworkers [13].

CEL files were loaded in the R-Bioconductor (BioC) software and normalized with the Robust Multi Chip Average (RMA) method [41]. Identification of differentially expressed genes for pairwise comparisons were performed using the RankProd R-package, which is based on the Rank Product principle [10]. We used the GoHyperG function from the BioC project to find over-represented biologic process GO categories from the gene lists using hypergeometric test for significance. KEGG pathway analysis was performed with the Webgestalt web interface using hypergeometric test for significance [42].

Meta-analyses of published neuroblastoma microarray data were performed with the NBGS (see Additional data fiile 3 for detailed information).

Positional gene enrichment analysis was performed with in-house developed R-Bioconductor script PGE (De Preter and coworkers, unpublished data) [15]. PGE scans the entire genome using a moving window with a user-defined width (5 Mb) and step size (1 Mb). In each window, the -10log(p) of the Fisher Exact Test is calculated. This test was used to investigate whether there is an association between the gene list and a particular chromosomal region (the window under investigation). As such, it will identify regions that contain more (or less) genes in the gene list than expected by chance. The (known) unequal distribution of the genes along the chromosomes is taken into account, because the number of genes from the list that are located in the region is compared with the total number of genes in that particular region. Correction for multiple testing is performed using the false discovery rate method of Benjamini and Hochberg [43], using the R-multtest package.

Expression microarray data were submitted to ArrayExpress [44], accession number E-MEXP-669.

## Additional data files

The following additional data are available with the online version of this paper. Additional data file 1 includes documents on RNA quality and quantity measures, validation of Affymetrix chip results, and Affymetrix chip quality parameters [39,45-49]. Additional data file 2 lists genes that are differentially expressed in neuroblast versus cortex samples, in neuroblast versus favorable (F) and/or unfavorable (UF) neuroblastoma, and in favorable versus unfavorable neuroblastoma (identified using Rank Product algorithm). Additional data file 3 provides results of NBGS analysis of the genes that are differentially expressed between neuroblasts and (favorable and/or unfavorable) neuroblastomas (gene lists in Additional data file 1). Additional data file 4 lists the genes of GO class neurogenesis and proneural genes that are expressed in neuroblast samples (> 36th percentile) [50-54].

## Authors' contributions

KDP performed the neuroblast microdissection and microarray data mining, and drafted the paper. PH collected the fetal adrenal glands and helped with the neuroblast microdissection. NY helped with microdissection, RNA isolation and quantification, RNA quality control and real-time quantitative polymerase chain reaction validation experiments. SB performed the immunohistochemical stainings that were reviewed and discussed by SP. AS, AE, RS, MR, YB, and GL collected neuroblastoma tumor samples. JV and FS participated in the study's design and coordination. All authors have reviewed the manuscript, and FS and ADP were the final editors of the manuscript.

## Supplementary Material

Additional data file 1 RNA quality and quantity measures, validation of Affymetrix chip results, and Affymetrix chip quality parameters Click here for file

Additional data file 2 Genes that are differentially expressed in neuroblast versus cortex samples, in neuroblast versus favorable (F) and/or unfavorable (UF) neuroblastoma, and in favorable versus unfavorable neuroblastoma Click here for file

Additional data file 3 Results of NBGS analysis of the genes that are differentially expressed between neuroblasts and (favorable and/or unfavorable) neuroblastomas (gene lists in Additional data file 1) Click here for file

Additional data file 4 Genes of GO class neurogenesis and proneural genes that are expressed in neuroblast samples (> 36th percentile) Click here for file

## Figures and Tables

**Table 1:** GO analysis: neuroblast versus cortex samples

GO	*P *value	*n*	GO description
More highly expressed in neuroblast compared to cortex			

GO:0007399	5.94E-09	20	Neurogenesis
GO:0019226	1.12E-06	13	Transmission of nerve impulse
GO:0007268	5.39E-06	12	Synaptic transmission
GO:0001505	9.29E-05	5	Regulation of neurotransmitter levels
GO:0007267	2.76E-04	16	Cell-cell signaling
GO:0050877	3.17E-04	16	Neurophysiological process
GO:0046879	3.47E-04	3	Hormone secretion
GO:0006584	3.47E-04	3	Catecholamine metabolism
GO:0018958	4.72E-04	3	Phenol metabolism
GO:0048513	6.67E-04	22	Organ development
GO:0006836	6.96E-04	4	Neurotransmitter transport
GO:0009887	7.32E-04	21	Organogenesis
GO:0007154	1.21E-03	46	Cell communication
GO:0045055	1.25E-03	3	Regulated secretory pathway
GO:0007269	1.25E-03	3	Neurotransmitter secretion
GO:0046903	1.52E-03	8	Secretion
GO:0030072	2.09E-03	2	Peptide hormone secretion
GO:0030073	2.09E-03	2	Insulin secretion
GO:0016079	2.09E-03	2	Synaptic vesicle exocytosis
GO:0042423	2.09E-03	2	Catecholamine biosynthesis
GO:0006887	2.24E-03	4	Exocytosis
GO:0009653	2.77E-03	23	Morphogenesis
GO:0007218	3.25E-03	4	Neuropeptide signaling pathway
GO:0007275	3.26E-03	29	Development
GO:0046883	5.70E-03	2	Regulation of hormone secretion
GO:0030182	7.26E-03	2	Neuron differentiation
GO:0048489	8.98E-03	2	Synaptic vesicle transport

More highly expressed in cortex compared to neuroblast			

GO:0016126	4.18E-07	5	Sterol biosynthesis
GO:0006694	9.28E-07	6	Steroid biosynthesis
GO:0008202	4.32E-06	7	Steroid metabolism
GO:0016125	2.93E-05	5	Sterol metabolism
GO:0044255	6.74E-05	9	Cellular lipid metabolism
GO:0006629	8.75E-05	10	Lipid metabolism
GO:0008610	1.39E-04	6	Lipid biosynthesis
GO:0006695	2.97E-04	3	Cholesterol biosynthesis
GO:0006066	1.17E-03	6	Alcohol metabolism
GO:0008203	4.69E-03	3	Cholesterol metabolism
GO:0044242	4.97E-03	2	Cellular lipid catabolism
GO:0006118	7.90E-03	5	Electron transport

Shown are over-represented GO classes (biological process) (with *P *< 0.01 and at least two genes) in the list of genes that are more highly expressed in neuroblast than in cortex samples, and *vice versa*. GO, Gene Ontology.

**Table 2:** GO analysis: normal neuroblasts compared to neuroblastomas

GO	*P *value	*n*	GO description
More highly expressed in neuroblast compared to neuroblastoma			

GO:0008202	1.33E-18	17	Steroid metabolism
GO:0006694	3.22E-15	12	Steroid biosynthesis
GO:0044255	2.13E-12	18	Cellular lipid metabolism
GO:0008610	8.54E-12	13	Lipid biosynthesis
GO:0016125	1.29E-11	10	Sterol metabolism
GO:0006629	1.50E-10	18	Lipid metabolism
GO:0008203	5.85E-09	8	Cholesterol metabolism
GO:0008207	1.37E-08	5	C21-steroid hormone metabolism
GO:0006700	1.37E-08	5	C21-steroid hormone biosynthesis
GO:0006066	1.55E-08	12	Alcohol metabolism
GO:0042446	9.26E-07	5	Hormone biosynthesis
GO:0016126	1.16E-06	5	Sterol biosynthesis
GO:0042445	9.57E-06	5	Hormone metabolism
GO:0006118	1.76E-05	9	Electron transport
GO:0006869	1.84E-05	5	Lipid transport
GO:0009058	4.62E-05	16	Biosynthesis
GO:0006695	5.39E-04	3	Cholesterol biosynthesis
GO:0042423	6.50E-04	2	Catecholamine biosynthesis
GO:0006810	2.29E-03	20	Transport
GO:0006091	2.32E-03	9	Generation of precursor metabolites and energy
GO:0006584	2.85E-03	2	Catecholamine metabolism
GO:0051234	2.88E-03	20	Establishment of localization
GO:0051179	3.04E-03	20	Localization
GO:0018958	3.46E-03	2	Phenol metabolism
GO:0042401	5.64E-03	2	Biogenic amine biosynthesis
GO:0042398	8.30E-03	2	Amino acid derivative biosynthesis

More highly expressed in neuroblastoma compared to neuroblast			

GO:0019882	4.54E-14	16	Antigen presentation
GO:0030333	2.51E-12	14	Antigen processing
GO:0019884	1.21E-08	8	Antigen presentation, exogenous antigen
GO:0019886	3.45E-08	8	Antigen processing, exogenous antigen via MHC class II
GO:0019883	4.23E-07	7	Antigen presentation, endogenous antigen
GO:0006260	2.07E-06	20	DNA replication
GO:0006955	2.45E-06	58	Immune response
GO:0006952	1.02E-05	60	Defense response
GO:0019885	1.03E-05	6	Antigen processing, endogenous antigen via MHC class I
GO:0009607	1.05E-05	66	Response to biotic stimulus
GO:0006270	9.25E-05	6	DNA replication initiation
GO:0006259	4.34E-04	35	DNA metabolism
GO:0009596	5.51E-04	4	Detection of pest, pathogen or parasite
GO:0006261	7.72E-04	10	DNA-dependent DNA replication
GO:0050896	1.03E-03	92	Response to stimulus
GO:0006913	1.42E-03	12	Nucleocytoplasmic transport
GO:0007051	1.84E-03	5	Spindle organization and biogenesis
GO:0016070	2.01E-03	25	RNA metabolism
GO:0007052	2.27E-03	4	Mitotic spindle organization and biogenesis
GO:0009595	2.27E-03	4	Detection of biotic stimulus
GO:0007017	2.67E-03	11	Microtubule-based process
GO:0006658	3.08E-03	2	Phosphatidylserine metabolism
GO:0009613	3.39E-03	34	Response to pest, pathogen or parasite
GO:0006928	3.94E-03	18	Cell motility
GO:0040011	3.94E-03	18	Locomotion
GO:0043207	4.72E-03	34	Response to external biotic stimulus
GO:0007626	5.10E-03	18	Locomotory behavior
GO:0016043	5.31E-03	46	Cell organization and biogenesis
GO:0016049	6.09E-03	12	Cell growth
GO:0008361	6.09E-03	12	Regulation of cell size
GO:0042254	6.39E-03	6	Ribosome biogenesis and assembly
GO:0051169	8.15E-03	10	Nuclear transport
GO:0016071	8.34E-03	16	Mrna metabolism
GO:0043241	8.91E-03	2	Protein complex disassembly
GO:0031498	8.91E-03	2	Chromatin disassembly
GO:0006337	8.91E-03	2	Nucleosome disassembly
GO:0006104	8.91E-03	2	Succinyl-coa metabolism
GO:0007610	9.44E-03	21	Behavior
GO:0007049	9.50E-03	39	Cell cycle

Shown are over-represented GO classes (biological process) (with *P *< 0.01 and at least two genes) in the list of genes that are more highly expressed in normal neuroblasts than in neuroblastomas, and *vice versa*. GO, Gene Ontology.

**Table 3:** Differentially expressed transcription factors, neurogenesis, and apoptosis genes

	Transcription factor (GO:0003700)		Neurogenesis (GO:0007399)		Apoptosis (GO:0006915)	
	
	Gene name	Location	Gene name	Location	Gene name	Location
Neuroblast > neuroblastoma	*MLL*	11q	*APOE*	19q	*APOE*	19q
	*NR0B1*	Xp	*GREM1*	15q	*PLAGL1*	6q
	*RORA*	15q	*TFAP2B*	6p	*SCARB1*	12q
	*TFAP2B*	6p			*TDGF1*	3p

Neuroblast < neuroblastoma	ASCL1*	12q	*ALK*	2p	*BCL2*	18q
	*ATF3*	1q	*APBB2*	4p	*BCLAF1*	6q
	*CNOT7*	8p	*ASCL1*	12q	*BIRC5*	17q
	*CUTL2*	12q	*CDK5R1*	17q	*CCL2*	17q
	*ETV6*	12p	*FEZ1*	11q	*CD2*	1p
	*FOXC1*	6p	*GPI*	19q	*CD74*	5q
	*HCLS1*	3q	*INHBA*	7p	*CIAPIN1*	16q
	*IRF8*	16q	*LARGE*	22q	*CYCS*	7p
	*KLF10*	8q	*MBNL1*	3q	*HTRA2*	2p
	*MLX*	17q	*NEFH*	22q	*IER3*	6p
	*NFE2L1*	17q	*NTRK3*	15q	*IGFBP3*	7p
	*NFIB*	9p	*OLFM1*	9q	*INHBA*	7p
	*NME2*	17q	*PPT1*	1p	*ITGB2*	21q
	*RUNX1*	21q	*SERPINF1*	17p	*ITGB3BP*	1p
	*SIX3*	2p	*SIX3*	2p	*LGALS1*	22q
	*STAT1*	2q	*SLIT1*	10q	*LY86*	6p
	*STAT3*	17q	*SOX11*	2p	*OPA1*	3q
	*TAF10*	11p	*STAT3*	17q	*PRKCA*	17q
	*TAF7*	5q	*TRAPPC4*	11q	*RNF130*	5q
	*TFDP1*	13q			*STAT1*	2q
	*TRIM22*	11p			*SULF1*	8q
	*TSC22D1*	13q			*TNFRSF21*	6p
	*ZNF91*	19p			*TUBB*	6p

Shown are differentially expressed genes in neuroblastoma versus neuroblasts that belong to GO terms transcription factor, neurogenesis, and/or apoptosis, with an indication of the chromosomal localization. GO, Gene Ontology.

**Table 4:** Genes that are differentially expressed in favorable vs. unfavorable neuroblastoma

Favorable NB > unfavorable NB		NBGS	Favorable NB < unfavorable NB		NBGS
	
neuroblast < favorable NB			neuroblast < favorable NB, neuroblast < unfavorable NB		
	
*AKAP7*	6q	-	*FABP6*	5q	-
*ARL7*	2q	-	*NEFL*	8p	-
*ASPN*	9q	-	*NPY*	7p	-
	
*BCL2*	18q	1	neuroblast < unfavorable NB		
	
*C2orf23*	2p	-	*ALK*	2p	-
*CALB1*	8q	-	*ASCL1*	12q	1
*CAMK2B*	7p	2	*BIRC5*	17q	3
*CD24*	6q	-	*C22orf18*	22q	-
*CDC42*	1p	1	*C3*	19p	-
*DDAH1*	1p	-	*CALCB*	11p	-
*DNAPTP6*	2q	-	*CCNB1*	5q	1
*EPB41L3*	18p	1	*CD74*	5q	-
*FAM70A*	Xq	-	*CRH*	8q	-
*KIFAP3*	1q	1	*CSPG3*	19p	-
*OPHN1*	Xq	-	*DNCI1*	7q	-
*PDLIM5*	4q	-	*DTL*	1	1
*PPAN*	19p	-	*F12*	5q	-
*PRKCB1*	16p	1	*GFRA2*	8p	-
*RGS7*	1q	2	*IGHG3*	14q	-
*RNF11*	1p	-	*IGHM*	14q	-
*ST6GALNAC5*	1p	-	*IGKC*	2p	1
*SV2C*	5q	-	*IGLC2*	22q	-

neuroblast > favorable NB, neuroblast > unfavorable NB			*LMO3*	12p	*1*

*CACNA2D3*	3p	-	*MGC27165*	14q	-
*DLK1*	14q	2	*MLF1IP*	4q	1
*HBG1*	11p	-	*MMP9*	20q	1
*HBG2*	11p	-	*MYCN*	2p	9

neuroblast > unfavorable NB			*NEFH*		

*ALDH3A2*	17p	1	*ODC1*	2p	3
*DLC1*	8p	-	*OGDHL*	10q	-
*EYA1*	8q	-	*P2RX5*	17p	-
*GCH1*	14q	1	*PRAME*	22q	1
*HBA2*	16p	-	*RPS4Y1*	Yp	1
*KIAA0960*	7p	1	*SERPINF1*	17p	-
*PTPRD*	9p	-	*TNFRSF10B*	8p	2
*PTPRK*	6q	-	*TOP2A*	17q	1
*SLC18A1*	8p	-	*TWIST1*	7p	1
*TFAP2B*	6p	-	*TYMS*	18p	-
*TLN2*	15q	1	*XAGE1*	Xp	-
	
			neuroblast > favorable NB, neuroblast < unfavorable NB		
	
			*IGLJ3*	22q	1
	
			neuroblast > favorable NB		
	
			*RNU2*	17q	-
	
			neuroblast > favorable NB, neuroblast > unfavorable NB		
	
			*LOC492304*	11p	-

Genes that are differentially expressed compared with neuroblasts among the differentially expressed genes in favorable neuroblastoma (NB) versus unfavorable neuroblastoma, with an indication of the number of neuroblastoma microarray studies in which these genes were found through NBGS analysis. NBGS, Neuroblastoma Gene Server.

**Table 5:** Clinical and genetic data of carefully selected neuroblastoma samples that were included in this study

Sample number	Lab number	% Tumor cells	Stage	*MYCN *amp	Ploidy	Adrenal localisation	Age	Dead/alive	Overall survival (months)	Type
NB1	01T15	80	4S	No	Tri	Yes	< 1 year	Alive	61.4	Favorable
NB2	98T33	95	1	No	Tri	Yes	< 1 year	Alive	76.9	Favorable
NB3	96T82	90	1	No	Tri	Yes	< 1 year	Alive	115.5	Favorable
NB4	99T129	90	1	No	Tri	Yes	< 1 year	Alive	71.7	Favorable
NB5	01T28	90	4	Yes	Di	Yes	> 1 year	Dead	5.6	Unfavorable
NB6	03T304	100	3^a^	No	Di	Abdominal	> 1 year	Alive	12.0	Unfavorable
NB7	03T236	90	4	No	ND	Yes	> 5 year	Dead	19.4	Unfavorable
NB8	00T54	70	1	No	Tri	Yes	< 1 year	Alive	62.6	Favorable
NB9	00T35	> 95	4	Yes	Di	Yes	< 1 year	Dead	13.7	Unfavorable
NB10	99T125	80	3	No	Di	Yes	> 5 year	Alive	79.3	Unfavorable
NB11	92W145	70	4	No	ND	ND	> 5 year	Dead	19.5	Unfavorable
NB12	02T192	100	4	Yes	Di	Abdominal	> 5 year	Dead	16.2	Unfavorable
NB13	D031	> 95	4	No	Di	Abdominal	> 1 year	Dead	64.8	Unfavorable
NB14	E002	> 80	4	No	ND	Abdominal	> 1 year	Alive	65.7	Unfavorable
NB15	E037	> 80	4	No	ND	Abdominal	> 1 year	Alive	45.3	Unfavorable
NB16	E044	> 80	4	No	ND	Yes	< 1 year	Alive	37.0	Unfavorable
NB17	E121	> 80	4	Yes	ND	Abdominal	> 1 year	Dead	78.4	Unfavorable
NB18	04T121	60	3	Yes	Di	Yes	> 1 year	Dead	6	Unfavorable

Based on stage, *MYCN *amplification, ploidy, and age at diagnosis, samples were subdivided into favorable or unfavorable type.

^a^Neuroblastoma or ganglioneuroblastoma. ND, not determined or unknown.
