# Supplementary material for: Human fetal neuroblast and neuroblastoma transcriptome analysis confirms neuroblast origin and highlights neuroblastoma candidate genes
Source: Genome Biol. 2006 Sep 21;7(9):R84. doi: 10.1186/gb-2006-7-9-r84 (PMC1794547; doi:10.1186/gb-2006-7-9-r84)
Supplement: Additional data file 1 — RNA quality and quantity measures, validation of Affymetrix chip results, and Affymetrix chip quality parameters [file gb-2006-7-9-r84-S1.doc]

ADDITIONAL DATA 1A:

In order to save the valuable RNA of the rare neuroblastic cells for downstream analyses, we used the abundantly available adjacent cortical cells from the same sections in order to assess RNA quality, assuming that the RNA of small islets of neuroblasts surrounded by these cells is equally preserved.

RNA was collected at every step of the process (after sectioning, H&E staining and RNA isolation) and assayed on the 2100 Bioanalyser capillary electrophoresis system (Agilent Technologies). The figure shows the total RNA quality profiles, RNA degradation coefficient (in %) [42] and 28S/18S ratio for unstained cryosections, H&E stained cryosections, microdissected cortical cells and microdissected neuroblasts (sample 3, isolate B). For the last sample, the amount of RNA was insufficient to determine ratio and degradation coefficient. The RNA degradation coefficients of the other samples show that RNA is only slightly degraded during the procedure (degradation coefficient going from 7.25%, over 7.56% to 13.85% for sample 3 isolate B). The fair RNA quality of the microdissected surrounding cortical cells allows us to assume similar RNA quality for the neuroblast samples for which insufficient amounts of RNA was available for reliable RNA quality analysis.


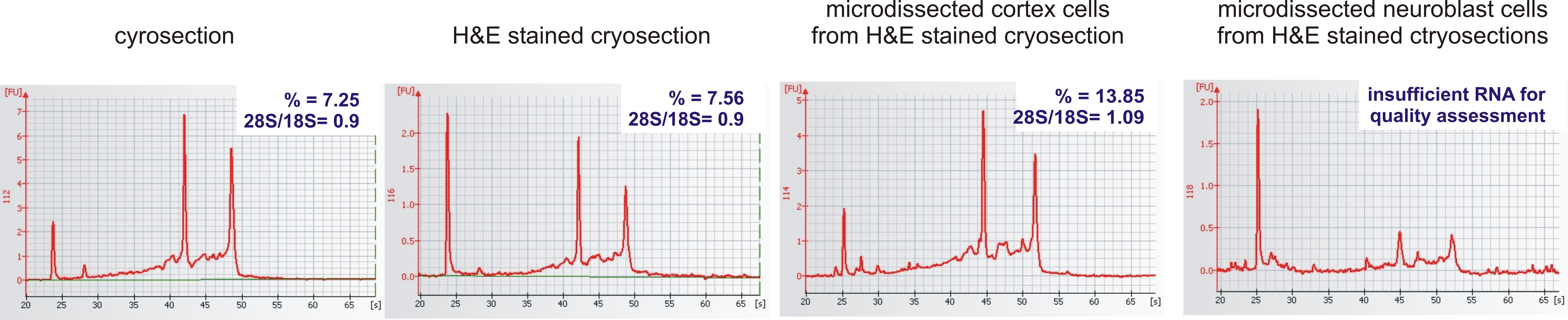


ADDITIONAL DATA 1B:

The RNA yield of microdissected surrounding cortical cells could be measured on a fluorometer as sufficient cells could be easily collected. In order to quantify the smaller amount of RNA from the foetal neuroblasts, real-time quantitative RT-PCR was performed. One twentieth of the total RNA of each neuroblast and cortex isolate was used for cDNA synthesis and real-time quantitative RT-PCR analysis of reference genes *UBC* and *GAPDH*. Based on a standard curve (3-point 10-fold serial dilution of fluorometrically quantified foetal adrenal cortex RNA), RNA concentrations of the microdissected neuroblast isolates were interpolated. For the cortex samples, a high correlation was observed between the RNA yields measured using real-time quantitative RT-PCR and fluorometric analysis. By pooling different isolates of the microdissected cells, between 2.5 and 15 ng of total RNA could be obtained for each neuroblast sample.

| sample number | cell type | **pg/µl (fluorometer)** | **pg/µl (based on geometric mean of UBC and GAPDH expression)** | **Estimated total amount of RNA (ng)** |
| --- | --- | --- | --- | --- |
| sample 1 | cortex | 5067.57 | 3803.79 | **57.06** |
| sample 1 isolate A | neuroblast | nd | 73.80 | **1.11** |
| sample 1 isolate B | neuroblast | nd | 17.00 | **0.26** |
| sample 1 isolate C | neuroblast | nd | 73.24 | **1.10** |
| sample 2 | cortex | 1258.01 | 1579.83 | **23.70** |
| sample 2 isolate A | neuroblast | nd | 117.14 | **1.76** |
| sample 2 isolate B | neuroblast | nd | 30.21 | **0.45** |
| sample 2 isolate C | neuroblast | nd | 102.10 | **1.53** |
| sample 2 isolate D | neuroblast | nd | 487.79 | **7.32** |
| sample 2 isolate E | neuroblast | nd | 151.42 | **2.27** |
| sample 2 isolate F | neuroblast | nd | 135.05 | **2.03** |
| sample 2 isolate G | neuroblast | nd | 40.60 | **0.61** |
| sample 3 | cortex | 807.5 | 1057.08 | **15.86** |
| sample 3 isolate A | neuroblast | nd | 102.95 | **1.54** |
| sample 3 isolate B | neuroblast | nd | 168.98 | **2.53** |
| sample 3 isolate C | neuroblast | nd | 270.34 | **4.06** |

### nd = not detectable

#### Methods

Depending on the number of cells, RNA concentration was determined by real-time quantitative RT-PCR or fluorometric analysis with PicoGreen according to the manufacturer’s protocol for low RNA concentration. For the real-time quantitative RT-PCR approach, a standard curve of cDNA with known concentration was run together with test cDNA for two reference genes, i.e. *GAPDH* and *UBC*. The mean of *GAPDH* and *UBC* results was a good estimate for the RNA concentration of the isolates.

ADDITIONAL DATA 1C:

RNA of neuroblast, cortex and neuroblastoma samples was hybridised to HG-U133A oligonucleotide chips after a two-round labelling and amplification protocol. To assess a potential amplification bias, the mRNA expression of seven genes (*CYP11A1*, *CYP17A1*, *DBH*, *DDC*, *GAP43*, *PHOX2A* and *PNMT*) was tested using real-time quantitative PCR in the neuroblast and cortex RNA samples before and after amplification. For 5 of the 7 genes the (Spearman) correlation was clearly significant (*CYP11A1* (p=0.111), *CYP17A1* (p=0.397), *DBH* (p=0.042), *DDC* (p=4.8E-3), *GAP43* (p=0.042), *PHOX2A* (p=0.016) and *PNMT* (p=0.019)).

In order to validate the chip expression levels, the expression level of *CYP11A1*, *CYP17A1*, *DBH*, *DDC*, *GAP43*, *PHOX2A* and *PNMT* was measured using qPCR in the neuroblast and cortex samples, and compared with the oligonucleotide chip data (see Table A). With the exception of 4 genes, a good correlation is observed between qPCR data and oligonucleotide chip data.

For 15 of the neuroblastoma samples, genes *DDC*, *GAP43*, *MYCN*, *NTRK1*, *PHOX2A* and *TH* were tested using qPCR: for each gene the Spearman correlation was significant at the 0.01 level (see Table B). The high correlation of the expression levels obtained with the two methods clearly shows that our oligonucleotide chip data based on two-round amplified material are reliable.

**Methods**

Primer pairs were constructed using Primer Express (Applied Biosystems). Primer sequences are available in the public RTPrimerDB database (<http://medgen.UGent.be/rtprimerdb/>): *CYP11A1* (3592), *CYP17A1* (3593), *DBH* (1086), *DDC* (365), *GAP43* (97), *MYCN* (11), *NTRK1* (118), *PHOX2A* (1085), *PNMT* (1087) and *TH* (3594) [44]. The relative expression levels of genes were determined using an optimized two-step real-time SYBR Green I RT-PCR assay [37] on the ABI5700 (Applied Biosystems). The gene expression levels were normalised using the geometric mean of reference genes (*GAPDH* and *UBC* in microdissected neuroblast and cortex samples and *GAPDH*, *HPRT1* and *UBC* in neuroblastoma samples and amplified neuroblast and cortex samples) as described previously [46].

ADDITIONAL DATA 1D:

In order to assess the quality of the hybridisation on the HG-U133A oligonucleotide chips after two-round RNA amplification/labelling, several technical quality parameters were evaluated. First of all, probe array images were shown to be free from artefacts (data not shown). In addition, standard array quality control metrics were evaluated through the simpleaffy package (R BioC) (based on MAS5 normalisation).

| **sample name** | percent present calls | average background | scale factor |
| --- | --- | --- | --- |
| *neuroblast 1* | 32.06 | 104.64 | 2.5 |
| *cortex 1* | 39.50 | 153.99 | 0.9 |
| *neuroblast 3* | 36.38 | 141.44 | 1.6 |
| *cortex 3* | 29.56 | 140.75 | 1.8 |
| *neuroblast 2* | 39.78 | 128.59 | 1.3 |
| *cortex 2* | 40.97 | 128.22 | 1.0 |
| *NB1* | 36.90 | 124.02 | 1.2 |
| *NB2* | 42.39 | 114.50 | 0.9 |
| *NB3* | 49.29 | 115.55 | 0.6 |
| *NB4* | 45.45 | 107.73 | 0.8 |
| *NB5* | 42.19 | 118.09 | 1.1 |
| *NB6* | 48.91 | 99.92 | 0.6 |
| *NB7* | 37.02 | 87.59 | 1.9 |
| *NB8* | 48.80 | 93.42 | 0.9 |
| *NB9* | 28.13 | 96.30 | 3.3 |
| *NB10* | 52.22 | 133.35 | 0.5 |
| *NB11* | 29.90 | 101.21 | 3.7 |
| *NB12* | 28.42 | 97.95 | 2.9 |
| *NB13* | 29.15 | 109.39 | 3.6 |
| *NB14* | 51.39 | 111.66 | 0.5 |
| *NB15* | 49.94 | 104.61 | 0.8 |
| *NB16* | 41.06 | 93.59 | 1.5 |
| *NB17* | 24.27 | 116.12 | 4.2 |
| *NB18* | 35.89 | 116.61 | 1.9 |

As shown in the table, comparable values were obtained for scale factor, average background and present call among the different chips. Except for two samples, the scale factors were within the 3-fold range. The average background values of all arrays were not within the 60-100 range, but are typically higher after application of the two-round labelling protocol. Percent present calls were between 25% and 50% for the different samples (with an average of 36%).

The application of a two-round protocol of RNA amplification with random hexamers leads to a more pronounced 3’ bias of the resulting antisense RNA transcripts, explaining the higher ratios of the 3’ probe set to the 5’ probe set for *GAPD* and *ACTB* (data not shown). Since the majority of probe sets on oligonucleotide chips represents the 3’ end of each transcript, this effect would not have a major impact on the quantification of most transcripts represented on the arrays as reported [43,45]. Moreover, the 3’ bias appears to be a general effect present in all chips justifying comparison of the different chips.
